# Personalized survival predictions via Trees of Predictors: An application to cardiac transplantation

**DOI:** 10.1371/journal.pone.0194985

**Published:** 2018-03-28

**Authors:** Jinsung Yoon, William R. Zame, Amitava Banerjee, Martin Cadeiras, Ahmed M. Alaa, Mihaela van der Schaar

**Affiliations:** 1 University of California Los Angeles, Los Angeles, California, United States of America; 2 Farr Institute of Health Informatics Research, University College, London, United Kingdom; 3 University of Oxford, Oxford, United Kingdom; 4 Alan Turing Institute, London, United Kingdom; Duke-NUS Medical School, SINGAPORE

## Abstract

**Background:**

Risk prediction is crucial in many areas of medical practice, such as cardiac transplantation, but existing clinical risk-scoring methods have suboptimal performance. We develop a novel risk prediction algorithm and test its performance on the database of all patients who were registered for cardiac transplantation in the United States during 1985-2015.

**Methods and findings:**

We develop a new, interpretable, methodology (ToPs: Trees of Predictors) built on the principle that specific predictive (survival) models should be used for specific clusters within the patient population. ToPs *discovers* these specific clusters and the specific predictive model that performs best for each cluster. In comparison with existing clinical risk scoring methods and state-of-the-art machine learning methods, our method provides significant improvements in survival predictions, both post- and pre-cardiac transplantation. For instance: in terms of 3-month survival post-transplantation, our method achieves AUC of 0.660; the best clinical risk scoring method (RSS) achieves 0.587. In terms of 3-year survival/mortality predictions post-transplantation (in comparison to RSS), holding specificity at 80.0%, our algorithm correctly predicts survival for 2,442 (14.0%) more patients (of 17,441 who actually survived); holding sensitivity at 80.0%, our algorithm correctly predicts mortality for 694 (13.0%) more patients (of 5,339 who did not survive). ToPs achieves similar improvements for other time horizons and for predictions pre-transplantation. ToPs discovers the most relevant features (covariates), uses available features to best advantage, and can adapt to changes in clinical practice.

**Conclusions:**

We show that, in comparison with existing clinical risk-scoring methods and other machine learning methods, ToPs significantly improves survival predictions both post- and pre-cardiac transplantation. ToPs provides a more accurate, personalized approach to survival prediction that can benefit patients, clinicians, and policymakers in making clinical decisions and setting clinical policy. Because survival prediction is widely used in clinical decision-making across diseases and clinical specialties, the implications of our methods are far-reaching.

## Introduction

Risk prediction (survival prediction) is crucial in many areas [1–3], perhaps most obviously in medical practice. Survival prediction before and after heart transplantation, which is the focus of this paper, is an especially important problem because transplantation and treatment decisions depend on predictions of patient survival on the wait-list and survival after transplantation [4, 5]. In addition to providing useful guidance for the treatment of individual patients, better predictions may increase the number of successful transplantations. (Currently, only about one-third of available hearts are transplanted; the other two-thirds are discarded [6–8]).

Risk/survival prediction in this context is a challenging problem for a number of reasons.

- The populations of patients and donors are heterogeneous. This heterogeneity is reflected in different survival patterns for subpopulations. Moreover, the importance of particular features (covariates) on survival is different for different sub-populations, and the dependence of survival on features involves the interactions *between* features, which are again different for different sub-populations.
- The features that have the most effect on survival depend on the time horizon: the features that are most important for survival for 3 months are different from those that are most important for survival for 3 years.
- The underlying clinical practice and the patient population change over time. In the case of heart transplantation, the most dramatic example of change in the underlying clinical practice took place following the introduction of mechanical assists, especially left ventricular assist devices (LVADs) in 2005, after which population average survival times improved significantly [9, 10].

Most of the commonly used clinical approaches to survival prediction use one-size-fits-all models that apply to the entire population of patients and donors and does not fully capture the heterogeneity of these populations. Most of these clinical approaches construct a single risk score (a real number) for each patient as a function of the patient’s features and then use that risk score to predict a survival time or a survival curve [11–16]. A consequence of this approach is that patients with higher risk are predicted to have a lower probability of surviving for every given time horizon—so survival curves for different individuals do not intersect.

The main objective of this study was to construct and test a new approach to survival problems: ToPs—trees of predictors. ToPs captures the heterogeneity of the populations by *learning, automatically on the basis of the data* which features have the most predictive power and which features have the most discriminative power *for each time horizon*. ToPs uses this knowledge to create *clusters of patients* and specific predictive models *for each cluster*. The clusters that are identified and the predictive models that are applied to each cluster are readily interpretable. ToPs can be easily re-trained to accommodate to changes in clinical practice and the patient/donor population. (A particularly dramatic change in clinical practice occurred in 2005 with the introduction of LVADs, which significantly improved patient survival. As we discuss below, when we re-trained ToPs on data that takes this change into account, predictive accuracy was also significantly improved.) We tested ToPs on the United Network for Organ Sharing database (using various time periods for training and testing) and found that it provided significantly better predictions of survival, both post- and pre-transplantation, than commonly used clinical approaches and state-of-the-art machine-learning methods.

## Materials

### Study design

We conducted our study using the United Network for Organ Sharing (UNOS) database of patients who were registered to undergo heart transplantation during the years from 1985 to 2015 (available at https://www.unos.org/data/). This provided a dataset of 59,820 patients who received heart transplants and 35,455 patients who were on a wait-list but did not receive heart transplants; we refer to the former as transplanted patients and to the latter as wait-listed patients. We excluded the patients whose age is less than 18 (to focus on adults); thus, our cohorts consist of 51,971 transplanted patients and 30,911 wait-listed patients. Of the 51,971 transplanted patients, 26,109 patients (50.2%) were followed until death, and the remaining 25,862 patients (49.8%) were right-censored (i.e., either still alive or their exact survival time was not known). Of the 30,911 wait-listed patients, 16,916 patients (54.7%) were followed until death; the remaining 13,995 patients (45.3%) were right-censored.

**Fig 1:**
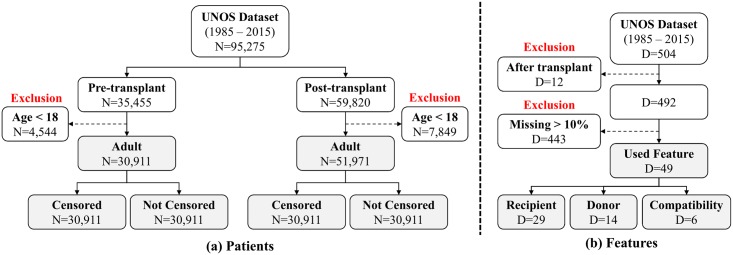
The summary of exclusion criteria for UNOS dataset. (a) Patients, (b) Features.

Patients in the dataset are described by a total of 504 clinical features. Of these, 334 features pertain to (potential) recipients, 150 features pertain to donors and 20 features pertain to donor-recipient compatibility. We discarded 12 features that can be obtained only after transplantation and so cannot be used for prediction before transplantation, and 439 features for which more than 10.0% of the information was missing. ([17] shows that imputing missing data is useful when the missing rate is less than 10.0%, but much less useful when the missing rate is higher.) After discarding these features, we were left with 33 recipient features, 14 donor features, and 6 donor-recipient compatibility features—a total of 53 features (See Supporting Information for the specific features used in this paper.) The exclusion criteria is summarized in Fig 1 for both patients and features.

Categorical binary features (e.g., Male/Female) are represented as 0, 1; categorical non-binary features are converted to binary features (e.g., blood type A/not-A); other features are represented as real numbers (Results from information theory imply that converting categorical non-binary features to multiple binary features is equivalent to using the original categorical non-binary features [18–20]; doing so is standard in data science [21, 22]). For missing components of the features, we use standard imputation methods to impute those components. More specifically, we conduct 10 multiple imputations using Multiple Imputation by Chained Equations (MICE) as in [17]. Furthermore, we assign a correlation coefficient between each feature and label to a relevance score for each feature. These scores depend on the task at hand: the relevance scores for survival on the wait-list are different for different time horizons, and the relevance scores for survival on the wait-list are different from the relevance scores for survival after transplantation (for various time horizons).

To develop and test our models we used 5-fold cross-validation. We randomly separated both the data set into 5 folds. In each of 5 iterations, 4 of the folds (80.0% of the data) were used for development of the models, and the remaining fold (20.0% of the data) was put aside for evaluating performance.

## Methods

### Model development: Personalization and clusters

As discussed in the Introduction, because patient (and patient-donor) features are heterogeneous, the predictive models to be used should be *personalized* to (the features of) a patient. We accomplish this personalization by using the data to identify *predictive clusters*—clusters of patients for whom predictions can best be made by using some specific learner, trained in some specific way (i.e. with coefficients fitted to some specific training set)—and then combining the predictions obtained over the clusters that contain the array of features of the particular patient for whom we need a prediction. This approach allows us to construct a complex predictive model from simple learners.

A potential problem with any such construction is that the predictive model becomes *too complex* and hence overfits. One way to control model complexity is to append a term that penalizes for complexity—but then the magnitude of the penalty term would become a parameter of the model. Instead, our approach controls model complexity *automatically* by training learners on one part of the development set and then validating on a different part of the development set. We give an outline below; further details, including the pseudo-code for the algorithm, can be found in the Supporting Information.

### Model development: Tree of predictors

Our method recursively splits the space *X* (of patient features or patient-donor compatibility features) into disjoint subsets (clusters) and, for each cluster, creates a specific predictive model by training a learner from the given base class *R* of learners (Cox Regression, Linear Perceptron, Logistic Regression) using the training set (the development set). This creates a *tree of predictors*: a tree *T* of clusters (*nodes*) of the feature space *X* together with a predictive model *h*_*C*_ associated to each cluster *C* in the tree. The overall prediction for a particular patient (or patient-donor pair) having a specific array of features is formed by finding the unique terminal node (cluster) of the tree to which that array of features belongs and the unique path through the tree from the initial node to this terminal node (this is precisely the set of clusters to which the patient belongs) and forming a weighted average of the predictions of the predictive models along this path, with weights determined (for the particular path) optimally by linear perceptron.

The construction of the optimal tree of predictors is recursive; at each stage, it builds on the tree previously constructed. We initialize the construction (Stage 0) by defining the initial node of the tree to be the entire space *X* of features. To determine the predictive model *h*_*X*_ to be assigned to the initial node, we train each of the learners in *R* globally; that is, for each learner, we find the parameters that create the predictive model that best fits the development set. Among these predictive models, we set *h*_*X*_ to be the one that yields the overall best fit. Having done this, we have built a (trivial) tree of predictors (i.e., a single node and predictive model). At each successive stage *n* + 1 we begin with the tree *T*_*n*_ of predictors constructed in previous stages. For each terminal node *C* of *T*_*n*_ we proceed as follows. Fix a feature *i* and a threshold *τ*_*i*_. (If necessary, we discretize all continuous features.) Define

$$C^{-}=\left\{x\in C:x_{i}<\tau_{i}\right\}\;\text{,}\;C^{+}=\left\{x\in C:x_{i}\geq \tau_{i}\right\}$$ (1)

Fix learners *L*^−^, *L*^+^ ∈ *R* and nodes *A*^−^, *A*^+^ ∈ *T*_*n*_ that weakly precede *C*^−^, *C*^+^, respectively. Train *L*^−^ on *A*^−^ (i.e., find the coefficients of *L*^−^ that best fit the portion of the development data with features in *A*^−^) and train *L*^+^ on *A*^+^; call the resulting predictive models *h*^−^, *h*^+^. Using *h*^−^ on *C*^−^ and *h*^+^ on *C*^+^ yields a predictive model *h*^−^ ∪ *h*^+^ on *C*^−^ ∪ *C*^+^ = *C*. Among all choices of the feature *i*, the threshold *τ*_*i*_, the learners *L*^−^, *L*^+^ and the training sets *A*^−^, *A*^+^, find those that maximize the AUC of *h*^−^ ∪ *h*^+^ on *C*. This defines a split of *C* = *C*^−^ ∪ *C*^+^ and predictive models associated with the sets *C*^−^, *C*^+^. If no further improvement is possible, we stop splitting. It can be shown that the process stops after some finite number of stages.

Note that at each stage the construction *jointly* chooses the feature, the threshold, the learner and the training set to *optimize the gain in predictive power* and the construction stops when no further improvement is possible; the end result of the process is the *optimal tree of predictors*.

**Fig 2:**
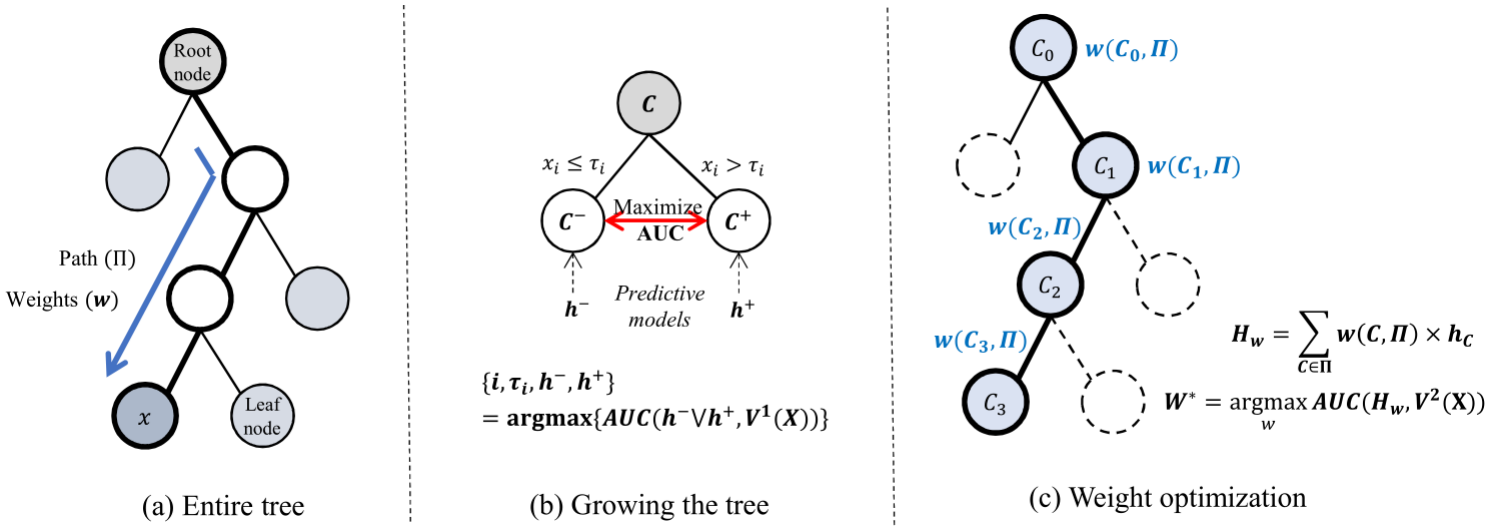
Illustration of ToPs construction.

To determine the prediction for a patient having the array *x* of features, we find the set Π of all nodes in the tree to which the array of features *x* belongs; this is the unique path from the initial node *X* to the unique terminal node to which *x* belongs. The overall prediction for *x* is the weighted average

$$\begin{array}{c} H\left(x\right)=\sum_{C\in \Pi }w\left(C,\Pi \right)\times h_{C}\left(x\right) \end{array}$$ (2)

where the weights *w*(*C*, Π) are determined optimally by linear regression. Fig 2 illustrate the construction of the tree of predictors.

**Fig 3:**
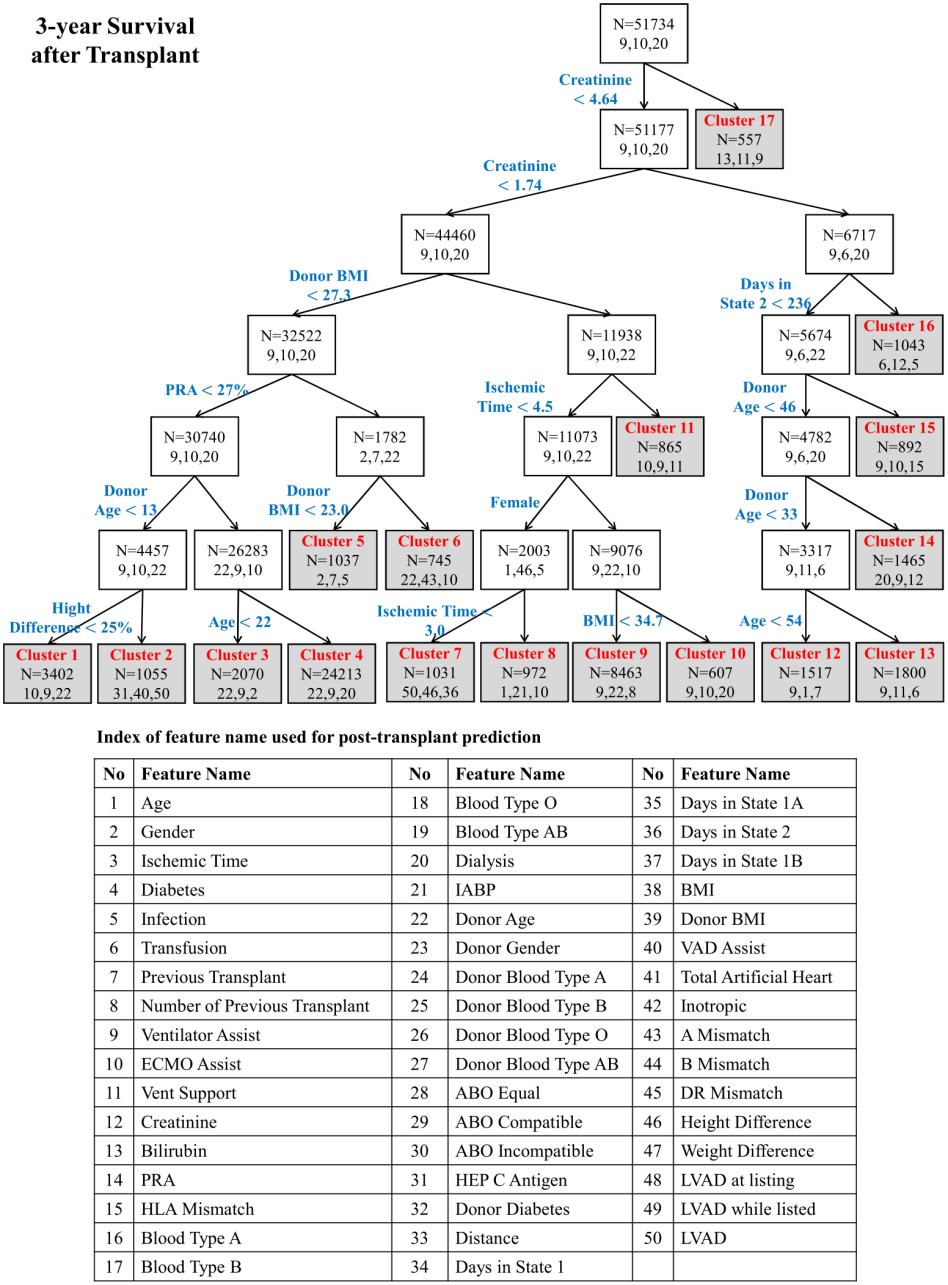
Tree of predictors for 3-year post transplantation survival (End nodes shaded gray. 3 most relevant features shown for each node as feature indices).

A typical optimal tree of predictors is shown in Fig 3, which shows the feature and threshold used to create each split. Within each cluster, it shows the total number of patients within that cluster and the index of the three most relevant features for that cluster. (The list of relevant features is shown in the lower part of Fig 3.) Note that, as is typical of our method, the tree constructed is not very deep—the recursive process of construction stops fairly quickly because no further improvement is possible. Consequently, only a few of these clusters are small, which helps to prevent overfitting.

### Implementation of ToPs

The implementation we use here uses as its base learners (algorithms) three familiar regression methods—Cox Regression, Linear Perceptron and Logistic Regression—so we use the acronym *ToPs/R*. In fact, we could use *any* collection of base learners and a larger or more sophisticated collection of base learners would lead to improved prediction accuracy. The choice of base learners made here was motivated by their familiarity and widespread use in the medical literature and because it lends itself more readily to interpretability of the clusters by clinicians.

### Clinical risk scores and survival prediction

Three predictive models for survival times post-transplantation are in widespread use in clinical practice: the Donor Risk Index (DRI), the Risk-Stratification Score (RSS), and the Index for Mortality Prediction After Cardiac Transplantation (IMPACT) [12, 13, 16]. We compare the post-transplantation predictions of our model with the predictions of these three clinical models. Similarly, three predictive models for survival times pre-transplantation (on the wait-list) are in widespread use in clinical practice: the Heart Failure Survival Score (HFSS), the Seattle Heart Failure Model (SHFM) and Meta-Analysis Global Group in Chronic Heart Failure (MAGGIC) [11, 14, 15]. We would have liked to compare the pre-transplantation predictions of our model with those of these three clinical models. Unfortunately, this was simply not possible because the UNOS dataset contains only 20% of the features used by these clinical models. All of the clinical predictive models compute an overall risk score—a weighted sum of scores of individual features. Patients with higher risk scores are viewed as more likely to die/less likely to survive for any given time horizon.

### Performance metrics and comparisons

The performance of any risk score can be assessed in terms of the *Receiver Operating Characteristic* (ROC) curve that plots the *True Positive Rate* (TPR) (*sensitivity*) against the *False Positive Rate* (FPR) (1—*specificity*). A summary performance statistic is the *Area Under the ROC Curve* (AUC). We report the performance (AUC for survival on the wait-list and for post-transplantation survival at the time horizons of 3 months, 1 year, 3 years and 10 years) of ToPs/R, of state-of-the-art machine learning methods, of regression methods, and of the relevant clinical risk scores. (When computing the AUC, we discard the censored patients whose survival time is less than the specific time horizon.) We also use the Concordance index (C-index) as another performance metric to evaluate the discrimination of the risk score [23]. We report the average C-index of 4 different time horizons. (In calculating the C-index, we do not discard any censored patients.) For evaluating the average population risks, we plot the calibration curve with 95% confidence bound.

## Results

### Performance improvement of ToPs/R

We evaluate the discriminative power of ToPs/R using both the C-index and AUC (at four different time horizons). Table 1 shows the predictive performance post-transplantation for ToPs/R, current clinical risk scores, familiar regression models and state-of-the-art machine learning benchmarks. Table 2 shows the predictive performance pre-transplantation (i.e., on the wait-list) for ToPs/R, familiar regression models, and state-of-the-art machine learning benchmarks. (In both settings, for the machine learning methods, we used 15% of the training samples for cross-validation in order to optimize hyper-parameters.) We do not compare with clinical risk scores pre-transplantation, because, as we have already noted, the UNOS dataset does not allow such comparisons. As can be seen, in comparison with other methods (including machine learning methods), our method provides consistent, large and statistically significant (p-value < 0.01) improvements in the prediction of survival both post- and pre-transplantation. The improvements over current clinical risk scores post-transplantation are particularly striking. For instance, for post-transplantation survival at a time horizon of 3 months, our method achieves AUC of 0.660 (95% Confidence Interval (CI): 0.650-0.671). By contrast, the best performing currently used clinical risk score (RSS) achieves AUC of only 0.587 (CI: 0.579-0.598). In terms of the C-index, our method achieves C-index of 0.577 (95% Confidence Interval (CI): 0.572-0.582). By contrast, the best performing currently used clinical risk score (RSS) achieves C-index of only 0.544 (CI: 0.539-0.549). The improvements achieved by our algorithm are similar for other time horizons.

**Table 1:** Comparisons among ToPs/R, existing clinical risk scores, regression methods, and machine learning benchmarks for post-transplantation survival prediction using C-index and AUC (at horizons of 3-months, 1-year, 3-years, and 10-years).

Methods	AUC (Mean ± Std)				C-index (Mean ± Std)
	3-month	1-year	3-year	10-year	
**ToPs/R**	**.660** ± **.003**	**.641** ± **.005**	**.623** ± **.005**	**.631** ± **.003**	**.577** ± **.003**
DRI	.540 ± .007	.547 ± .004	.547 ± .003	.556 ± .005	.529 ± .002
IMPACT	.561 ± .005	.556 ± .006	.549 ± .007	.558 ± .005	.527 ± .003
RSS	.587 ± .006	.582 ± .006	.570 ± .004	.547 ± .003	.544 ± .003
Cox	.572 ± .006	.579 ± .005	.553 ± .005	.577 ± .004	.519 ± .003
Linear P	.632 ± .007	.617 ± .003	.596 ± .003	.612 ± .005	.554 ± .003
Logit R	.629 ± .007	.613 ± .007	.599 ± .006	.611 ± .007	.554 ± .004
AdaBoost	.605 ± .006	.605 ± .006	.588 ± .004	.596 ± .004	.551 ± .003
DeepBoost	.594 ± .009	.608 ± .004	.591 ± .006	.594 ± .004	.548 ± .003
LogitBoost	.621 ± .005	.614 ± .004	.596 ± .004	.611 ± .003	.554 ± .003
XGBoost	.565 ± .007	.553 ± .005	.548 ± .003	.584 ± .005	.530 ± .003
DT	.592 ± .007	.595 ± .004	.575 ± .004	.595 ± .003	.543 ± .003
RF	.625 ± .004	.610 ± .004	.597 ± .003	.607 ± .004	.555 ± .003
NN	.600 ± .003	.608 ± .007	.587 ± .004	.598 ± .003	.550 ± .003

Linear P: Linear Perceptron, Logit R: Logistic Regression, DT: Decision Tree, RF: Random Forest, NN: Neural Nets

**Table 2:** Comparisons among ToPs/R, regression methods, and machine learning benchmarks for pre-transplantation survival prediction using C-index and AUC (at horizons of 3-months, 1-year, 3-years, and 10-years).

Methods	AUC (Mean ± Std)				C-index (Mean ± Std)
	3-month	1-year	3-year	10-year	
**ToPs/R**	**.685** ± **.003**	**.667** ± **.005**	**.652** ± **.009**	**.663** ± **.005**	**.603** ± **.003**
Cox	.624 ± .005	.623 ± .008	.614 ± .006	.612 ± .006	.534 ± .004
Linear P	.671 ± .004	.653 ± .002	.633 ± .006	.653 ± .009	.584 ± .003
Logit R	.672 ± .004	.651 ± .006	.635 ± .007	.650 ± .009	.582 ± .004
AdaBoost	.633 ± .004	.640 ± .008	.624 ± .007	.628 ± .009	.577 ± .004
DeepBoost	.635 ± .004	.645 ± .004	.626 ± .006	.620 ± .016	.578 ± .004
LogitBoost	.674 ± .006	.654 ± .008	.641 ± .009	.647 ± .006	.584 ± .004
XGBoost	.614 ± .005	.596 ± .007	.593 ± .007	.582 ± .010	.550 ± .004
DT	.664 ± .005	.646 ± .005	.618 ± .007	.610 ± .007	.574 ± .003
RF	.660 ± .004	.642 ± .004	.611 ± .007	.618 ± .009	.571 ± .003
NN	.637 ± .004	.641 ± .005	.629 ± .006	.622 ± .010	.580 ± .003

An alternative illustration of the improvement achieved by our method is the increase in the *number* of correctly predicted patients. For instance, for 3-year survival after transplantation, holding specificity at 80.0%, ToPs/R correctly predicted survival for 2,442 *more* patients (14.0% of the 17,441 patients survived up to 3 years) and, holding sensitivity at 80%, ToPs/R correctly predicted mortality for 694 *more* patients (13.0% of the 5,339 patients who died within 3 years), in comparison with the best clinical risk score, RSS. Because survival (or mortality) in the wait-list (urgency) and survival post-transplantation (benefit) are perhaps the most important factors in transplant policy, these predictive improvements are of great importance.

### Calibration of ToPs/R

**Fig 4:**
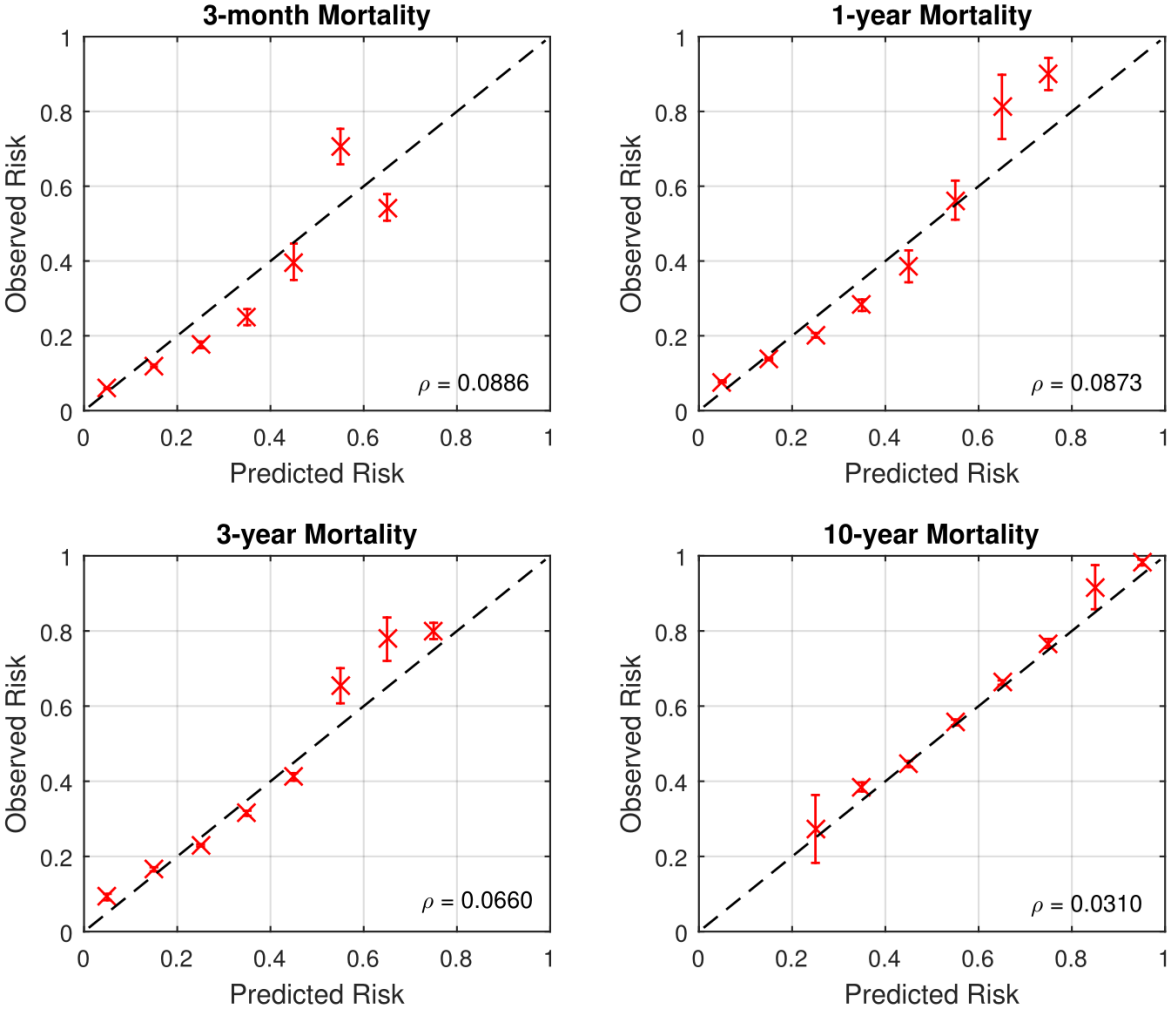
Calibration graphs for post-transplantation (*ρ* = Root mean square error).

**Fig 5:**
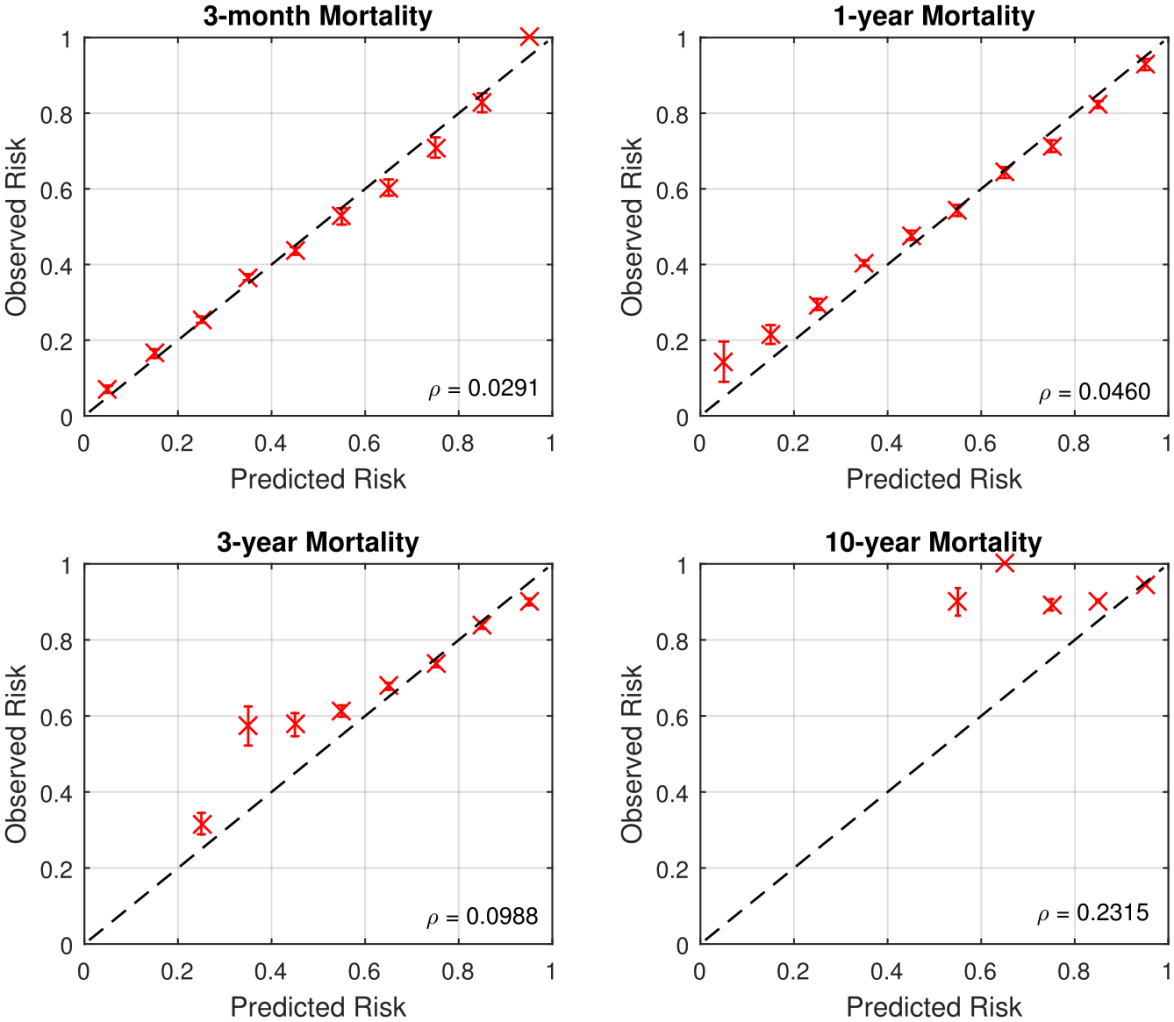
Calibration graphs for pre-transplantation (*ρ* = Root mean square error).

To evaluate the average performance across the population, we plot calibration graphs for each time horizon. The various panels of Figs 4 and 5 show calibration graphs for 3-month, 1-year, 3-year, and 10-year survival predictions, both post- and pre-transplantation. The value *ρ* represents the root mean square error between optimal calibration graph and the calibration graph of ToPs/R. It also shows the 95% confidence interval of observed risks for each calibration point.

As can be seen in Figs 4 and 5, in most cases the calibration graph of ToPs/R is closely aligned with the optimal calibration graph with small root mean-square error: *ρ* < 0.1. There are two cases in which the calibration graph of ToPs/R is less closely aligned with the optimal calibration graph. The first is for 10-year survival prediction pre-transplantation, where the mean square error is 0.2315; the second is for 3-month survival prediction post-transplantation, where the calibration graph does not capture high risk patients well. In both of these cases, the main problem is that the dataset is very unbalanced: the 10-year pre-transplantation survival rate is only 6.6%, and the 3-month post-transplantation mortality rate is only 9.2%. In the other settings, the dataset is much more balanced (e.g., the 3-month and 1-year pre-transplantation mortality rates are 19.7% and 33.9%, respectively) and the calibration graph is closely aligned to the optimal calibration graph, so that average predicted mortality is close to actual average mortality).

### Which features?

**Fig 6:**
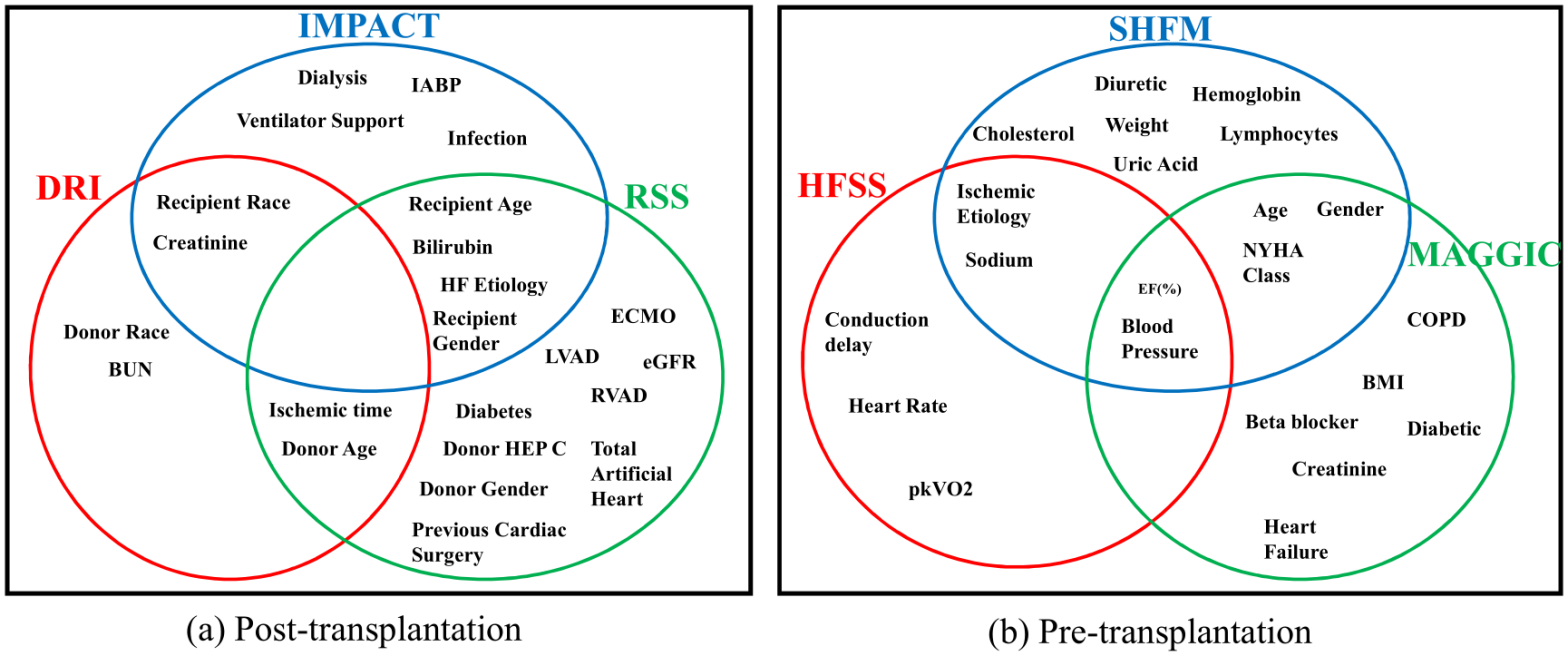
Venn diagram of clinical features used for each clinical risk score. (a) Post-transplantation, (b) Pre-transplantation.

A striking observatiodn about the leading post-transplantation clinical risk-scoring methods is that the three methods use quite different sets of clinical features. Indeed, as illustrated in Fig 6, there is *no one feature* that is used by all three clinical risk-scoring methods. This certainly suggests that there has been no clinical consensus about which features are important for evaluating post-transplantation risk. We have already noted that the UNOS dataset contains only 20% of the features used by the leading pre-transplantation clinical risk scores, and in fact, these methods also use rather different sets of features, so it seems that has been no clinical consensus about which features are important for evaluating pre-transplantation risk either.

Motivated by these observations, we evaluated the performance (using the concordance index) of ToPs/R, clinical risk scores, and the best ML benchmark for post-transplantation survival using various sets of features: (1) All *F*: all the 53 features used above, (2) Medical *F*: the 23 features used by at least one of the leading clinical risk scoring methods (IMPACT, DRI, and RSS) and (3) RSS *F*: the 14 features used by the best-performing clinical risk-scoring method (RSS). The results, are shown in Table 3. We highlight several findings and conclusions: (1) The performance of ToPs/R using all 53 features (All *F*) is indistinguishable (within margin of error) from its performance using only the 23 features used by at least one of the leading clinical risk-scoring methods (Medical *F*) but significantly better than the performance using only the 14 features used by RSS (RSS *F*). This suggests that, although no single clinical risk-scoring method seems to use all the most relevant features, when taken together, the three leading clinical risk-scoring methods *have* identified the most relevant features. And this shows that ToPs/R is capable of *discovering* the most relevant features. (We note that the leading ML method also seems to be capable of discovering the most relevant features.) (2) In all three settings, the performance of ToPs/R is superior to the performance of the best ML method and the best clinical risk-scoring method. Thus, ToPs/R makes *better use* of the given set of features—whichever set of features is given.

**Table 3:** Comparison of C-index among ToPs/R, existing clinical risk scores, and the best machine learning benchmark for survival prediction in post-transplantation with different feature sets.

Features	C-Index (Mean ± Std)		
	All *F*	Medical *F*	RSS *F*
**ToPs/R**	**.577 ± .003**	**.578 ± .003**	**.569 ± .002**
DRI	.529 ± .002	.529 ± .002	.529 ± .002
IMPACT	.527 ± .003	.527 ± .003	.527 ± .003
RSS	.544 ± .003	.544 ± .003	.544 ± .003
Best ML	.555 ± .003	.558 ± .003	.548 ± .002

*F*: Features, ML: Machine learning benchmark.

### Changing clinical practice

**Fig 7:**
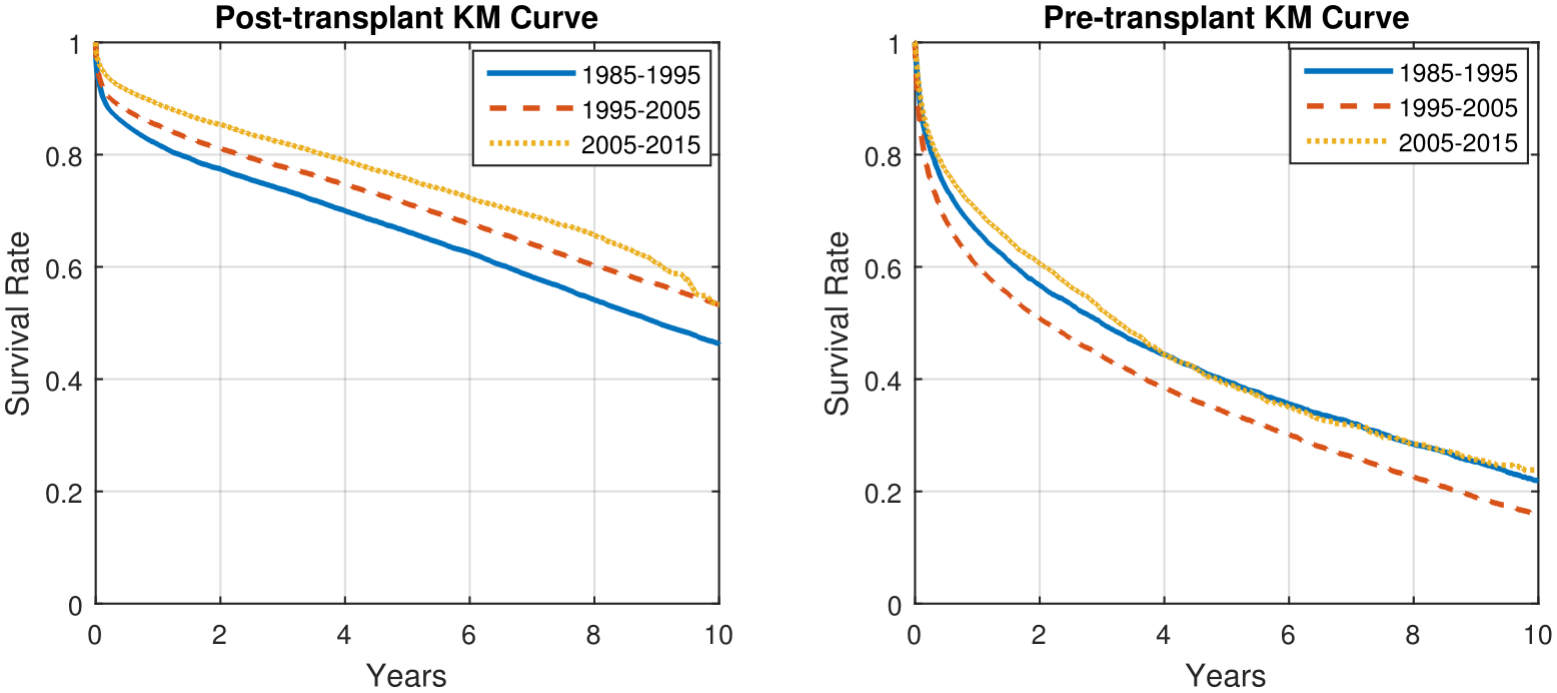
Kaplan-Meier survival curves for different periods in post- and pre- transplantation.

Clinical practice changes over time. In particular, the treatment of heart failure changed dramatically with the introduction of LVADs in 2005 [9, 10, 24–26], but there were also other, less dramatic changes during the period 1985-2015. Because of changes in selection criteria and availability of donor organs, the patient population also changed over this period, but perhaps less dramatically. As can be seen in Fig 7, the population-level Kaplan-Meier survival curves for the periods 1985-1995, 1995-2005, and 2005-2015 reflect these changes. (The pre-transplantation survival curve for 1995-2005 is below the pre-transplantation survival curve for 1985-1995; this may reflect different selection criteria rather than differences in treatment protocols.) It should be expected that as clinical practice, the patient population and the actual survival change, predictability should also change. Of course, it is not possible for ToPs/R—or any other method—to predict the effect of an improvement in clinical practice until data following that change is available. However, once such data is available, ToPs/R can use that data to provide improved predictions.

To illustrate this point, we use data from 2005-2009 (the first five-year period following the widespread introduction of LVADs) to predict 3-month, 1-year and 3-year survival both pre- and post-transplantation in the period 2010-2015. The results, shown in Tables 4 and 5, which should be compared with Tables 1 and 2, show that ToPs/R can utilize recent data to greatly improve the accuracy of prediction when treatment protocols (or patient populations) change. For example, the AUC for 3-month post-transplantation survival prediction increases from 0.660 when training on 1985-2015 and testing on 1985-2015 to 0.688 when training on 2005-2009 and testing on 2010-2015. Even more dramatically, the AUC for 3 month post-transplantation survival prediction increases from 0.685 (training on 1985-2015 and testing on 1985-2015) to 0.758 (training on 2005-2009 and testing on 2010-2015). This improvement in predictive accuracy reflects the facts that treatment protocols *did* change significantly over the 30-year period 1985-2015. In particular, it seems clear that widespread introduction of LVADs in 2005 made a significant difference in treatment protocols and hence in survival—and also in predictability. As can be seen in Tables 4 and 5, the model achieves significantly higher predictive accuracies when trained on patients in 2005-2009(recent patients) and tested on patients in 2010—2015 (current patients) than when trained and tested on patients in 1985-2015.

**Table 4:** Comparisons among ToPs/R, existing clinical risk scores, regression methods, and machine learning benchmarks for post-transplantation survival prediction using C-index and AUC (at horizons of 3-months, 1-year, and 3-years). Train on 2005-2009; predict on 2010-2015.

Methods	AUC (Mean ± Std)			C-index (Mean ± Std)
	3-month	1-year	3-year	
**ToPs/R**	**.688** ± **.001**	**.651** ± **.001**	**.639** ± **.009**	**.625** ± **.007**
DRI	.551 ± .014	.559 ± .016	.546 ± .014	.542 ± .013
IMPACT	.598 ± .013	.593 ± .001	.585 ± .011	.574 ± .009
RSS	.593 ± .017	.599 ± .020	.584 ± .013	.580 ± .012
Cox	.588 ± .012	.581 ± .009	.560 ± .010	.565 ± .007
Linear P	.666 ± .018	.632 ± .009	.600 ± .008	.608 ± .008
Logit R	.662 ± .009	.633 ± .007	.604 ± .008	.609 ± .006
AdaBoost	.643 ± .009	.630 ± .009	.606 ± .013	.607 ± .009
DeepBoost	.643 ± .009	.630 ± .010	.608 ± .006	.606 ± .008
LogitBoost	.655± .009	.632 ± .007	.602 ± .013	.607 ± .008
XGBoost	.574 ± .010	.567 ± .011	.554 ± .010	.555 ± .008
DT	.603 ± .009	.619 ± .008	.575 ± .009	.585 ± .007
RF	.641 ± .009	.628 ± .006	.613 ± .008	.606 ± .006
NN	.648 ± .014	.628 ± .009	.600 ± .011	.604 ± .007

**Table 5:** Comparisons among ToPs/R, regression methods, and machine learning benchmarks for pre-transplantation survival prediction using C-index and AUC (at horizons of 3-months, 1-year, and 3-years). Train on 2005-2009; predict on 2010-2015.

Methods	AUC (Mean ± Std)			C-index (Mean ± Std)
	3-month	1-year	3-year	
**ToPs/R**	**.758** ± **.008**	**.746** ± **.001**	**.746** ± **.026**	**.685** ± **.005**
Cox	.614 ± .010	.620 ± .007	.630 ± .011	.579 ± .006
Linear P	.734 ± .009	.728 ± .010	.722 ± .017	.668 ± .007
Logit R	.734 ± .008	.728 ± .007	.731 ± .013	.670 ± .005
AdaBoost	.718 ± .006	.699 ± .008	.706 ± .017	.644 ± .005
DeepBoost	.726 ± .008	.701 ± .009	.702 ± .015	.646 ± .007
LogitBoost	.736 ± .006	.727 ± .008	.719 ± .015	.667 ± .005
XGBoost	.646 ± .007	.630 ± .009	.616 ± .013	.586 ± .006
DT	.706 ± .007	.673 ± .007	.655 ± .016	.620 ± .005
RF	.721 ± .010	.705 ± .007	.669 ± .016	.644 ± .006
NN	.724 ± .007	.706 ± .011	.706 ± .012	.647 ± .007

### Predictive features

**Fig 8:**
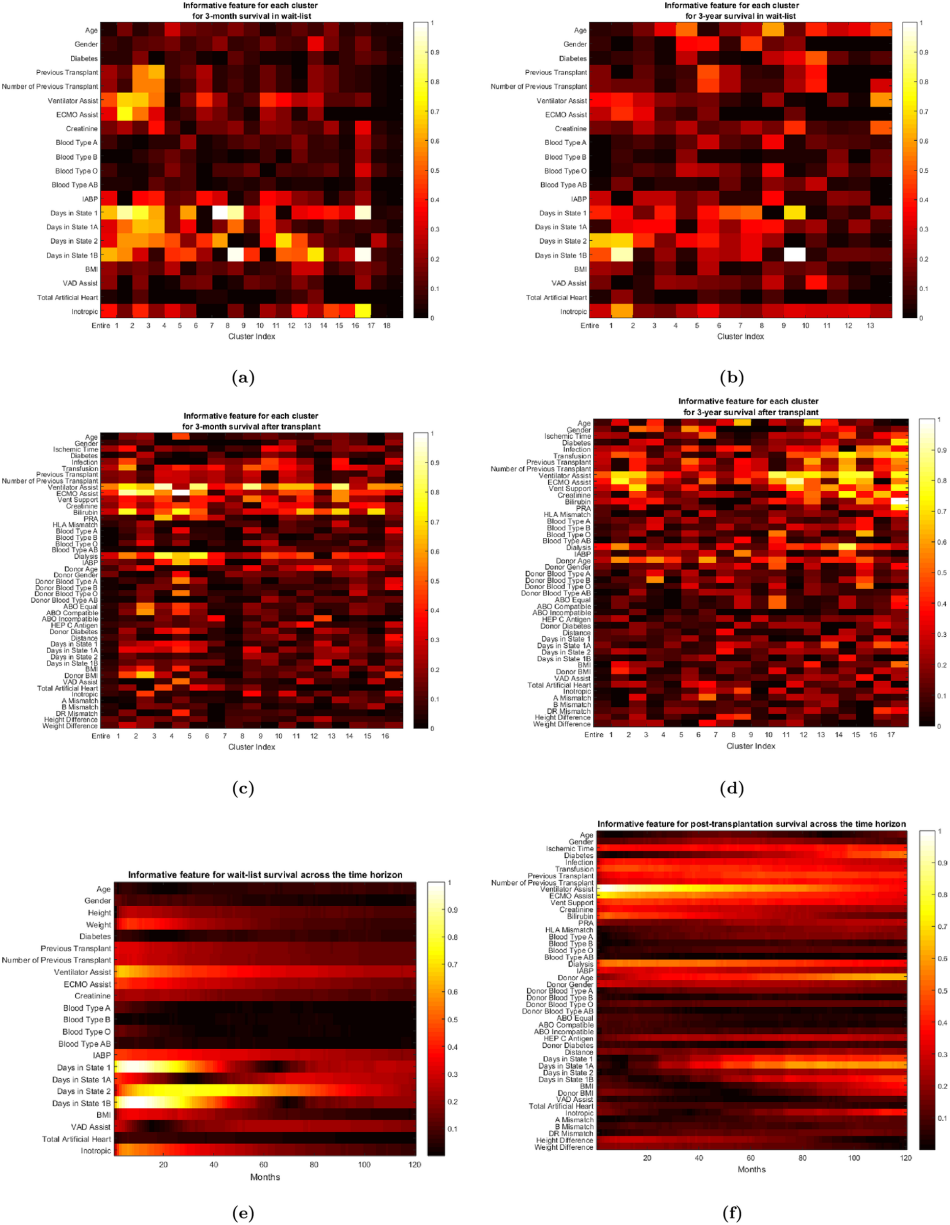
(a) Informative features for each cluster for 3-month pre-transplantation survival prediction, (b) 3-year pre-transplantation survival prediction, (c) for 3-month post-transplantation survival prediction, (d) for 3-year post-transplantation survival prediction, (e) for pre-transplantation survival across the time horizon, (f) for post-transplantation survival across the time horizon. (a ∼ f: from top left to bottom right).

Some features are more predictive of survival than other features, and some features are more predictive for survival for a particular time horizon than for other time horizons. Fig 8 presents heat maps displaying the predictive value of the features for various predictions over different time horizons. For instance, as can be seen in Fig 8(f), donor’s age is a very predictive feature for long-term post-transplantation survival, but it is less predictive of short-term survival; the most predictive features for short-term survival are the need for advanced cardiac and respiratory life support (ECMO and ventilator support, etc.). Moreover, the predictive power of donor’s age also differs across various sub-populations as well as across different time horizons. For instance, as Fig 3 shows, ToPs/R splits the entire population according to whether the creatinine level is below/above 4.64 mg/dL. The set of patients for whom the creatinine level is above 4.64 mg/dL is deemed Cluster 17; ToPs/R does not split this cluster any further. To see the importance of features in Cluster 17, refer again to the heat map Fig 8(d): we see that comorbidities such as diabetes are much more predictive of long-term survival than the donor’s age. This is consistent with the fact that chronic kidney disease and its interrelated set of comorbidities (which includes diabetes) can generally worsen cardiovascular outcomes [27, 28]. Diabetes does not have the same predictive power for other groups of patients. For example, in Cluster 5 (patients with creatinine level below 1.74 mg/dL, Panel Reactive Antibody (PRA) above 27% and BMI within the “normal” range 23.0-27.3 kg/m, the donor’s age and the ischemic time have more predictive power than diabetes. In this particular example, creatinine serves as a discriminative feature that filters out populations for whom survival prediction needs to consider different predictive features via “customized” predictive models. This example also sheds light on how ToPs/R can recognize the impact of comorbidities—in this case, renal failure and diabetes—on a patient’s survival: ToPs/R recognizes some features related to comorbidities as being discriminative, and also learns the appropriate predictive features for patients with these comorbidities. Note that two of the clinical risk scores—Donor Risk Index (DRI) and IMPACT—do not use diabetes as an input to their prediction rule; this may lead to misinformed surgical decisions for patients with comorbidities [12, 13].

## Discussion

In this study, we develop a methodology for personalized prediction of survival for patients with advanced heart failure while on the wait-list and after heart transplantation. Our methods and associated findings are important because they outperform the clinical risk scores currently in use and also because they automate the discovery and application of cluster-specific predictive models and tune predictions to the specific patient for which the prediction is to be made. Moreover, our predictive model can be easily and automatically re-trained as clinical practice changes and new data becomes available. The clinical and public health implications of our findings are broad and include improved personalization of clinical assessments, optimization of decision making to allocate limited life-saving resources and potential for healthcare cost reduction across a range of clinical problems.

### Key findings

We emphasize three key findings of our study:

- Our method significantly outperforms existing clinical risk scores, familiar regression models, and state-of-the-art machine learning benchmarks in terms of accurate prediction of survival on the wait-list and post-transplantation.
- This improvement in performance has clinical significance: our method correctly predicts both survival and mortality for a larger number of patients.
- There is substantial heterogeneity—both across clusters of patients and across different time horizons. Our method captures this heterogeneity far better than other methods.

### The sources of gains for ToPs/R

As the results show, ToPs/R achieves large performance improvements over current clinical risk-scoring models. It does so by explicitly addressing the weaknesses of these clinical models:

- Tops/R addresses the *heterogeneity of the population(s)* by identifying sub-populations (clusters) and the specific predictive models that are best suited to prediction in each sub-population. Tops/R makes predictions that are *personalized* to the features of the patient (or patient and donor).
- Tops/R addresses the *interactions between features* by using non-linear predictive models for those sub-populations (clusters) in which the interactions between features are important.
- Tops/R addresses the *heterogeneity across time horizons* by constructing different predictions for different time horizons.

Using different predictive models on different clusters means that we allow different features to have different importance and to interact differently for different clusters of patients. And because we make the choice of predictive models endogenously and optimally, and update as clinical practice changes and new data becomes available *we are letting the data tell us which choices to make*.

As we have already noted, ToPs/R finds the most relevant features *and* uses those features more effectively.

### Non-proportional and heterogeneous hazards models

The existing clinical risk scores do not produce individual survival curves. Clinicians using these scores infer survival curves by clustering patients with similar scores and constructing Kaplan-Meier curves from the actual survival times for these clusters. Our method produces individual survival curves by interpolating the predictions for 3 months, 1 year, 3 years and 10 years. Our approach can be viewed as providing a non-parametric survival model. In terms of hazard functions, our method could be interpreted as forming clusters, assigning a model to each cluster, and then aggregating those models to construct a non-proportional hazard function. In contrast to familiar approaches [29, 30] our approach *learns* which clusters to create, which models to assign and how to aggregate these models.

Cox Regression does produce individual survival curves. However, because Cox Regression assumes that relative hazard rates are constant over time, the survival curves produced by Cox Regression for two different patients cannot cross: if the survival probability for patient 1 is greater than that of patient 2 at a time horizon of 3 months it will also be greater at every time horizon. However, actual survival curves *may cross* [31, 32]. Our method allows for this—and this is a *virtue* because it reflects the fact that the features and interactions that are most important for survival at 3 months are different from the features and interactions that are most important for survival at longer horizons. (See Fig 8).

### Clinical support

Our work provides support for clinical decision making *in real time*. By using our user-friendly website http://medianetlab.ee.ucla.edu/ToPs_TransplantSurvival and entering relevant features, the clinician can obtain immediate predictions for a specific patient, including survival on the wait-list, the impact of an LVAD (if relevant), and benefit of transplantation (if relevant). All of this can be done at the desk of the clinician or the bedside of the patient, in no more time than is currently required to access the clinical risk scores, and with much greater accuracy (and confidence).

### Risk scoring and usability

Our analysis shows that our method provides significantly greater predictive power for survival while on the wait-list and post-transplantation. However, more research in actual practice is required. This success of our method in the setting of cardiac transplantation suggests it may have wide applicability and usability for risk prognosis and diagnosis for other medical conditions and diseases. The methodology developed here can also be applied in other settings, in particular to transplantation of other organs, such as kidneys; we leave this for future work. Such wider applications may benefit from further refinements of our method.

### Limitations

The results of this study carry limitations associated with the quality of the source data and the amount of missing data. Moreover, is not possible to integrate the effect of changes in treatment protocols until sufficient time has elapsed and sufficient new data becomes available. However, once sufficient data has become available, our method can integrate the new data to provide improved predictions. (See Tables 4 and 5, where we use data from the first 5 years of the LVAD era to make more accurate predictions for the following 5 years).

Our method produces risk scores and survival prediction both for patients both pre- and post-transplantation. We have provided extensive performance comparisons with the post-transplantation predictions of clinical and machine learning methods and with the pre-transplantation predictions of machine learning risk methods. As we have noted, it is unfortunately it is not possible to provide meaningful pre-transplantation comparisons with existing clinical methods such as HFSS, SHFM, and MAGGIC [11, 14, 15] because UNOS does not collect many of the features on which these clinical risk scores rely, such as ejection fraction, etc.

## Conclusion

We offer a new method for personalized risk prediction using a novel machine learning technique. Our method is explicitly designed to address the heterogeneity of the patients/donor populations by identifying various sub-populations. It captures the different effects of features and interactions *between* features for these various sub-populations. It also captures the different effects of features and interactions between features for different time horizons. Our method outperforms existing clinical scores and state-of-the-art machine learning methods both pre- and post-transplantation and for different time horizons. Moreover, our method is easily interpretable and applicable by clinicians. The present study has important clinical implications for the practice and policy of heart transplantation. The general methodology developed here has wide applicability to the construction of personalized risk scores in other medical domains.

## Supporting information

S1 Table Features used in ToPs/R. (PDF)

S2 Table Features used in medical scores for post-transplantation. (PDF)

S3 Table Abbreviation. (PDF)

S1 Algorithm Pseudo-code of ToPs/R. (PDF)

S1 Fig Tree of predictors for the post-transplantation survival (3-month) discovered by ToPs/R. (TIF)
