# Supplementary material for: Personalized survival predictions via Trees of Predictors: An application to cardiac transplantation
Source: PLoS One. 2018 Mar 28;13(3):e0194985. doi: 10.1371/journal.pone.0194985 (PMC5874060; doi:10.1371/journal.pone.0194985)
Supplement: S1 Table — (PDF) [file pone.0194985.s001.pdf]

S1 Table. Features used in ToPs/R

Recipient		Donor	Compatibility
Age	Blood Type AB	Age	ABO Compatibility
Gender	Dialysis	Gender	Ischemic time
Height	IABP	Height	HLA Mismatch
Weight	Days in State 1	Weight	A Mismatch
Diabetes	Days in State 1A	Blood Type A	B Mismatch
Infection	Days in State 2	Blood Type B	DR Mismatch
Transfusion	Days in State 1B	Blood Type O	
Previous Tx	BMI	Blood Type AB	
of Previous Tx	LVAD Assist	HEP C Antigen	
Ventilator Assist	Total Artificial Heart	Diabetes	
ECMO Assist	Inotropic	BMI	
Circulatory Assist	EF	Infection	
Creatinine	pk VO2	Bilirubin	
Bilirubin	ACE inhibitor	Creatinine	
PRA	Beta Blocker		
Blood Type A	Blood Type B		
Blood Type O			
