# Supplementary material for: Personalized survival predictions via Trees of Predictors: An application to cardiac transplantation
Source: PLoS One. 2018 Mar 28;13(3):e0194985. doi: 10.1371/journal.pone.0194985 (PMC5874060; doi:10.1371/journal.pone.0194985)
Supplement: S2 Table — (PDF) [file pone.0194985.s002.pdf]

S2 Table. Features used in medical score for post-transplantation

DRI	RSS	IMPACT
Ischemic Time	Recipient Age	Recipient Age
Donor Age	Previous Cardiac Surgery	Total Bilirubin
Recipient Race	Etiology	Creatinine
Donor Race	Diabetes complicated by CVA	Dialysis in listing
BUN	eGFR	Recipient Gender
Creatinine	Total Bilirubin	Total Bilirubin
	Acuity (Intubated / Hospitalized)	Heart Failure Etiology
	RVAD only Support	Infection
	ECMO Support	IABP Support
	Extracorporeal LVAD Support	Mechanical Ventilator Support
	Total Artificial Heart Support	Recipient Race
	Paracorporeal LVAD Support	
	Donor HEP C	
	Insulin Dependent Donor	
	Donor Age (years)	
	Ischemic Time (hours)	
	Donor Gender	
	Recipient Gender	
