# Supplementary material for: Personalized survival predictions via Trees of Predictors: An application to cardiac transplantation
Source: PLoS One. 2018 Mar 28;13(3):e0194985. doi: 10.1371/journal.pone.0194985 (PMC5874060; doi:10.1371/journal.pone.0194985)
Supplement: S3 Table — (PDF) [file pone.0194985.s003.pdf]

S3 Table. Abbreviations

No	Abbreviation	Meaning
1	ACE	Angiotensin-converting Enzyme
2	ACEI	Angiotensin-converting Enzyme Inhibitor
3	ARB	Angiotensin Receptor Blocker
4	AUC	Area Under the Curve
5	BMI	Body Mass Index
6	BNP	B-type Natriuretic Peptide
7	BUN	Blood Urea Nitrogen
8	COPD	chronic obstructive pulmonary disease
9	CVA	Cerebrovascular Accident
10	DRI	Donor Risk Index
11	ECMO	Extracorporeal Membrane Oxygenation
12	EF	Ejection Fraction
13	eGFR	Estimated Glomerular Filtration Rate
14	HEP	Hepatitis
15	HFSS	Heart Failure Survival Score
16	HLA	Human leukocyte antigen
17	IABP	Intra-Aortic Balloon Pump
18	IMPACT	Index for Mortality Prediction After Cardiac Transplantation
19	LVAD	Left Ventricular Assist Device
20	LVEF	Left Ventricular Ejection Fraction
21	MAGGIC	Meta-Analysis Global Group in Chronic Heart Failure
22	NYHA	New York Heart Association
23	pkVO2	Peak Oxygen Consumption by Cardiopulmonary Exercise Testing
24	PRA	Panel Reactive Antibody
25	RSS	Recipient Stratification Score
26	RVAD	Right Ventricular Assist Device
