# Supplementary material for: Personalized survival predictions via Trees of Predictors: An application to cardiac transplantation
Source: PLoS One. 2018 Mar 28;13(3):e0194985. doi: 10.1371/journal.pone.0194985 (PMC5874060; doi:10.1371/journal.pone.0194985)
Supplement: S1 Algorithm — (PDF) [file pone.0194985.s004.pdf]

S1 Algorithm. Pseudo-code of ToPs/R

Off-line Stage 0: Dividing the dataset (D)

Input: Entire dataset D
Divide D into disjoint training set (S), the first validation set (V^1), the second validation set (V^2) and testing set (T) which satisfy $D = S \cup V^1 \cup V^2 \cup T$
Output: Training set S, validation sets V^1, V^2, and testing set T

Off-line Stage 1: Growing the Optimal Tree of Predictors

Input: Feature space X, a set of algorithms $\mathcal{A}$, training set S, the validation set V^1
First step: Initial tree of predictors = (X, h_X) where $h_X = \arg\min_{A \in \mathcal{A}}$
Recursive step:
Input: Current tree of predictors $(T, \{h_C\})$
for each terminal node $C \in T$ **do**
  for a feature i and a threshold τ_i **do**
    Set $C^-(\tau_i) = \{x \in C : x_i < \tau_i\}, C^+(\tau_i) = \{x \in C : x_i \geq \tau_i\}$,Then,
    $\{i^*, \tau_i^*, h_{C^-(\tau_i^*)}, h_{C^+(\tau_i^*)}\} = \arg\min \mathcal{L}(h^- \cup h^+, V^1(C^-(\tau_i)) \cup V^1(C^+(\tau_i)))$
    where $h^- \in A(C^-(\tau_i)^\uparrow), h^+ \in A(C^+(\tau_i)^\uparrow)$
Stopping Criteria: $\mathcal{L}(h_C, V^1(C)) \leq \min \mathcal{L}(h^- \cup h^+, V^1(C^-(\tau_i)) \cup V^1(C^+(\tau_i)))$
Output: Locally optimal tree of predictors $(T, \{h_C\})$

Off-line Stage 2: Weights Optimization on the Path

Input: Locally optimal tree of predictors $(T, \{h_C\})$, the second validation set V^2
for each terminal node $\bar{C}$ and the corresponding path Π from X to $\bar{C}$ **do**
  for each weight vector $w = (w_C)$, **do**
    Define $H_w = \sum_{C \in \Pi} w_C h_C$, Then,
    $w^*(\Pi) = (w^*(\Pi, C)) = \arg\min \mathcal{L}(H_w, V^2(\bar{C}))$
  Output: Optimized weights $w^*(\Pi)$ for each terminal node $\bar{C}$ and corresponding path Π

On-line Stage: Overall Predictor

Input: Locally optimal tree of predictors $(T, \{h_C\})$, optimized weights $w^*(\Pi)$, and testing set T
Given a feature vector x
Find the unique path $\Pi(x)$ from X to terminal node containing x
Then, $H(x) = \sum_{C \in \Pi(x)} w^*(\Pi, C) h_C(x)$
Output: The final prediction $H(x)$
