# Supplementary figures and images for: Personalized survival predictions via Trees of Predictors: An application to cardiac transplantation
Source: PLoS One. 2018 Mar 28;13(3):e0194985. doi: 10.1371/journal.pone.0194985 (PMC5874060; doi:10.1371/journal.pone.0194985)

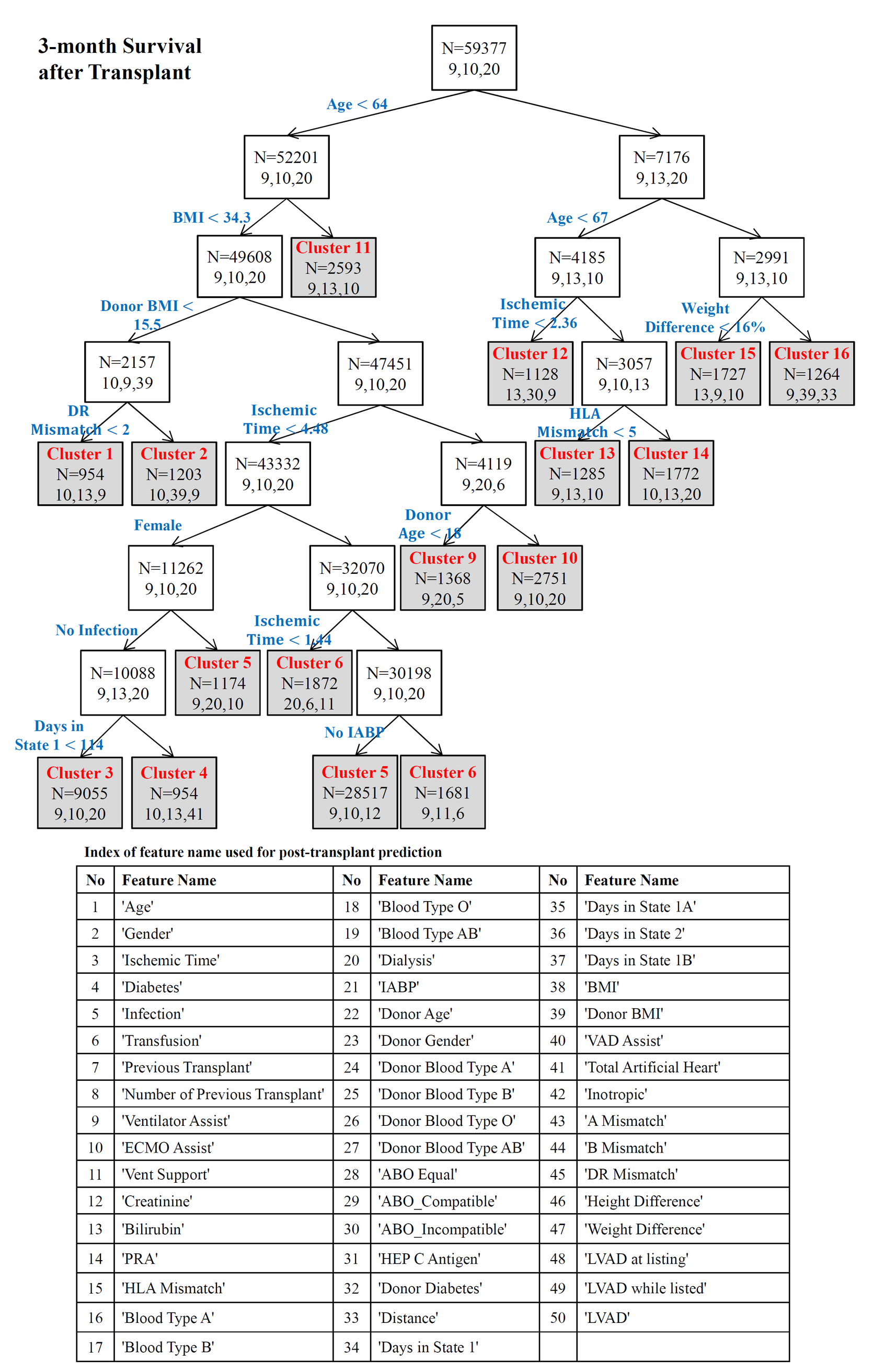

Supplement: S1 Fig — (TIF) [file pone.0194985.s005.tif]
